# Supplementary figures and images for: Possibility of deterioration of respiratory status when steroids precede antiviral drugs in patients with COVID-19 pneumonia: A retrospective study
Source: PLoS One. 2021 Sep 2;16(9):e0256977. doi: 10.1371/journal.pone.0256977 (PMC8412353; doi:10.1371/journal.pone.0256977)

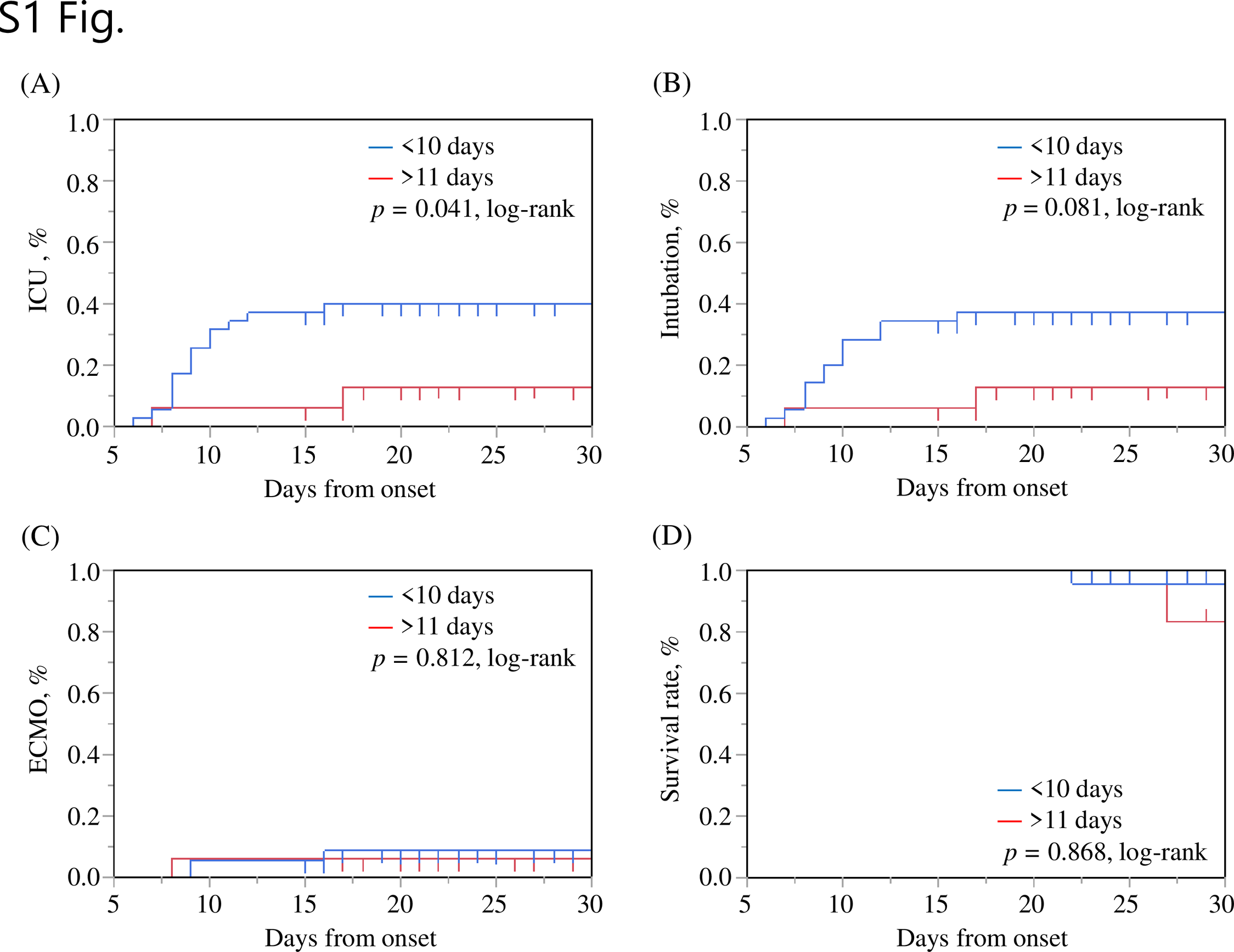

Supplement: S1 Fig — There are the tendencies in which the rate of ICU admission, intubation was poor in patients who administered steroid before 10 days. ECMO, Extracorporeal membrane oxygenation. ICU, Intensive care unit. (TIF) [file pone.0256977.s001.tif]

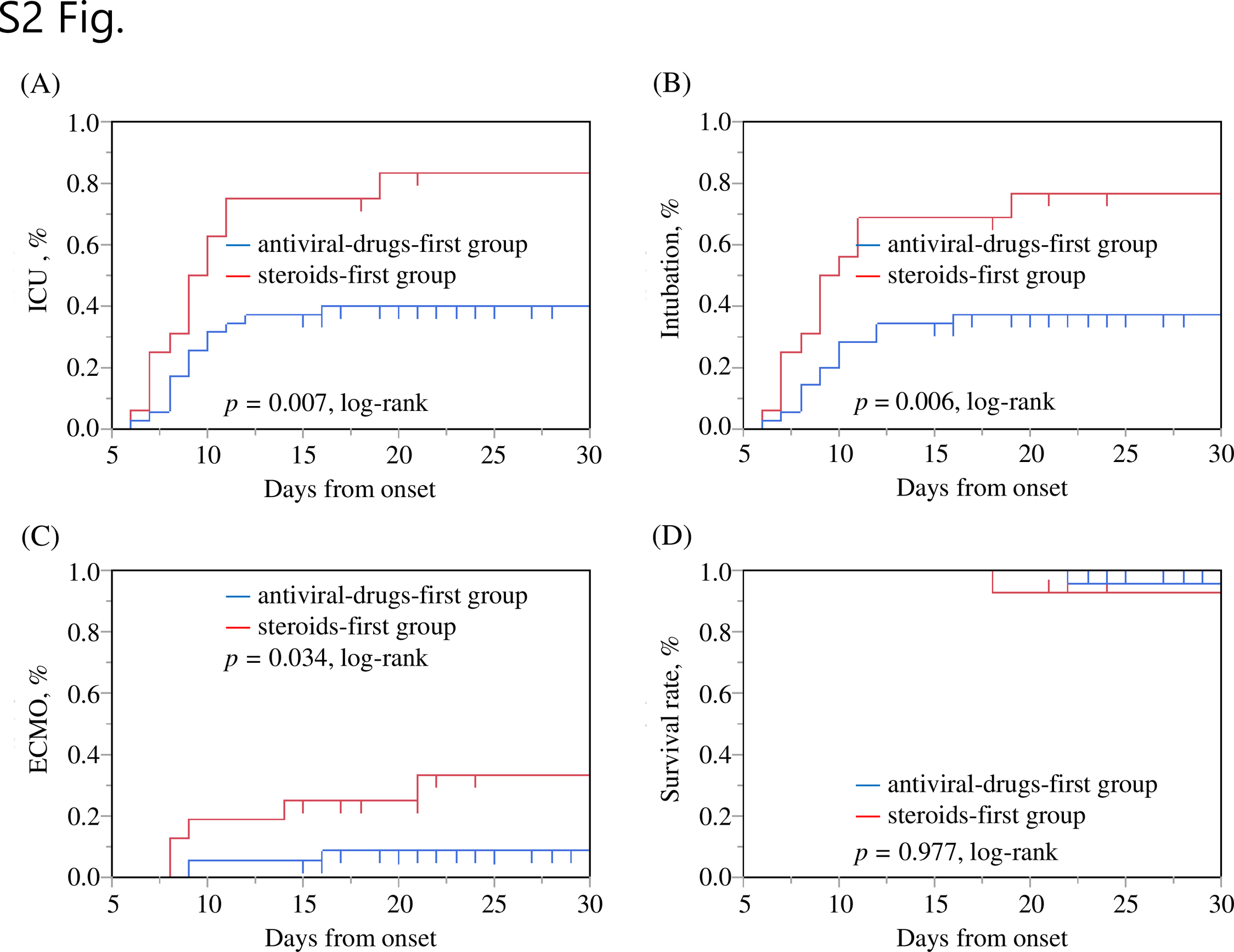

Supplement: S2 Fig — The rate of ICU admission, intubation, ECMO induction were poorer in patients of the steroids-first group than in those the antiviral-drugs-first group (p = 0.007, p = 0.007 and p = 0.037, respectively, log-rank test). ECMO, Extracorporeal membrane oxygenation. ICU, Intensive care unit. (TIF) [file pone.0256977.s002.tif]

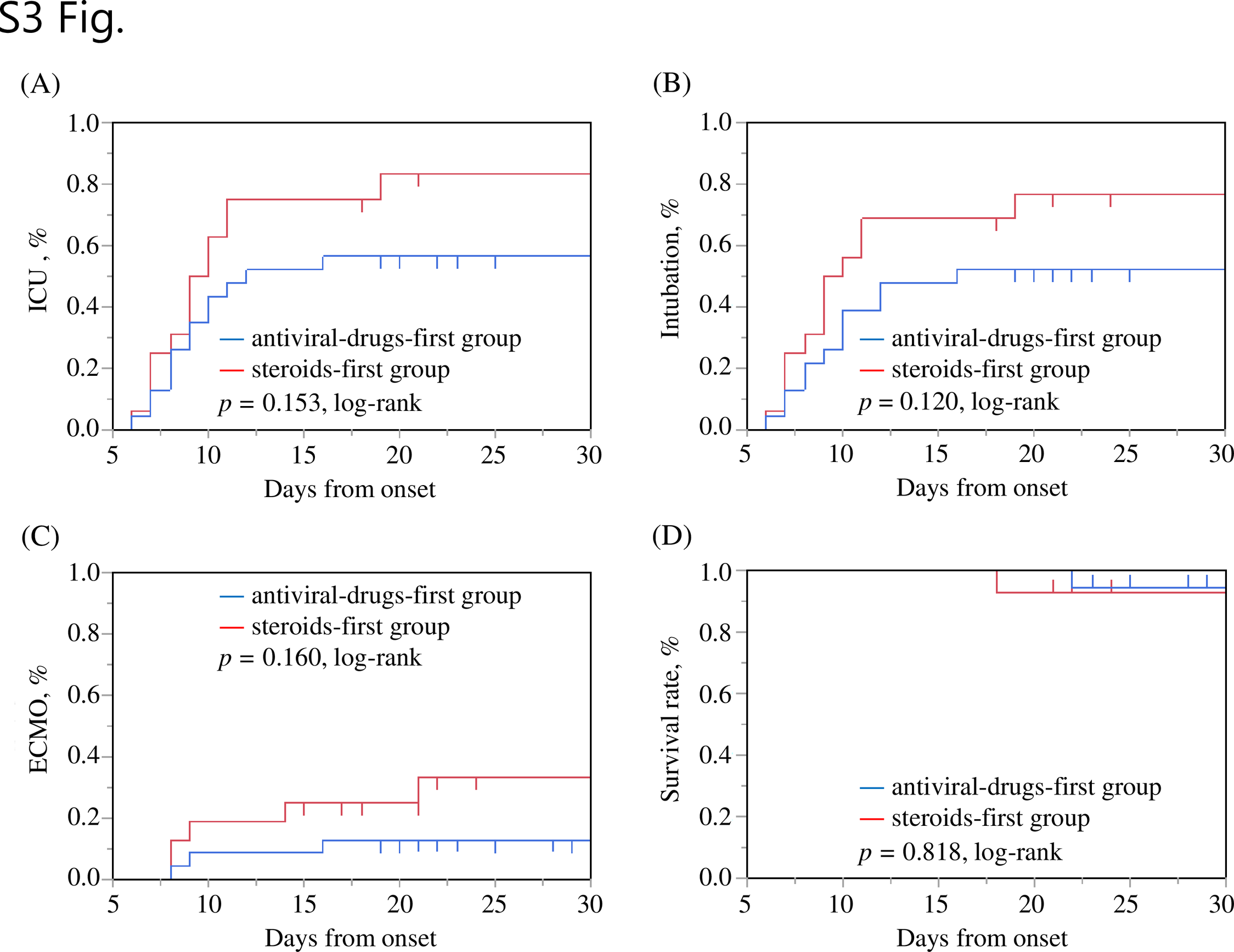

Supplement: S3 Fig — There are the tendencies in which the rate of ICU admission, intubation, ECMO induction were poor in patients of steroids-first group. ECMO, Extracorporeal membrane oxygenation. ICU, Intensive care unit. (TIF) [file pone.0256977.s003.tif]
