# Supplementary material for: Possibility of deterioration of respiratory status when steroids precede antiviral drugs in patients with COVID-19 pneumonia: A retrospective study
Source: PLoS One. 2021 Sep 2;16(9):e0256977. doi: 10.1371/journal.pone.0256977 (PMC8412353; doi:10.1371/journal.pone.0256977)
Supplement: S1 Table — (DOCX) [file pone.0256977.s004.docx]

**S1 Table.** Comparison between the group in whom steroids administered before 10 days and after 11 days in the antiviral-drugs-first group

| Parameter | Before 10 days  (n=35) | After 11 days  (n=16) | P-value |
| --- | --- | --- | --- |
| ICU admission, n (%) | 15 (42.9) | 2 (12.5) | **0.033** |
| Intubation, n (%) | 13 (37.1) | 2 (12.5) | 0.073 |
| ECMO, n (%) | 3 (8.6) | 1 (6.3) | 0.775 |
| Mortality at 30 days, n (%) | 5 (14.3) | 2 (12.5) | 0.864 |

ECMO, Extracorporeal membrane oxygenation. ICU, Intensive care unit.
