# Supplementary material for: Possibility of deterioration of respiratory status when steroids precede antiviral drugs in patients with COVID-19 pneumonia: A retrospective study
Source: PLoS One. 2021 Sep 2;16(9):e0256977. doi: 10.1371/journal.pone.0256977 (PMC8412353; doi:10.1371/journal.pone.0256977)
Supplement: S2 Table — (DOCX) [file pone.0256977.s005.docx]

**S2 Table.** Comparison between the antiviral-drugs-first group and the steroids-first group in cases where steroids were administered before 10 days of onset

| Parameter | antiviral-drugs-first group  (n=35) | steroids-first group  (n=16) | P-value |
| --- | --- | --- | --- |
| ICU admission, n (%) | 15 (42.9) | 13 (81.3) | **0.011** |
| Intubation, n (%) | 13 (37.1) | 12 (75.0) | **0.012** |
| ECMO, n (%) | 3 (8.6) | 5 (31.3) | **0.039** |
| Mortality at 30 days, n (%) | 5 (14.3) | 3 (18.8) | 0.684 |

ECMO, Extracorporeal membrane oxygenation. ICU, Intensive care unit.
