# Supplementary material for: Possibility of deterioration of respiratory status when steroids precede antiviral drugs in patients with COVID-19 pneumonia: A retrospective study
Source: PLoS One. 2021 Sep 2;16(9):e0256977. doi: 10.1371/journal.pone.0256977 (PMC8412353; doi:10.1371/journal.pone.0256977)
Supplement: S3 Table — (DOCX) [file pone.0256977.s006.docx]

**S3 Table.** The time difference between antiviral drugs administration to dexamethasone administration

| Parameter | **steroids-first group**  **(N=16)** | **antiviral-drugs-first group (N=51)** | | |
| --- | --- | --- | --- | --- |
|  |  | within 24 hours  (n=33) | within 48 hours  (n=40) | within 72 hours  (n=44) |
| ICU admission, n (%) | 13 (81.3) | 13 (39.4) ^†^ | 16 (46.9) ^†^ | 16 (36.4) ^†^ |
| Intubation, n (%) | 12 (75.0) | 11 (33.3) ^†^ | 14 (35.0) ^†^ | 14 (31.8) ^†^ |
| ECMO, n (%) | 5 (31.3) | 3 (9.1) * | 3 (7.5) * | 3 (6.8) * |

ECMO, Extracorporeal membrane oxygenation. ICU, Intensive care unit.

versus Steroids-proceeded group, * P<0.05, † P<0.01
