# Supplementary material for: Possibility of deterioration of respiratory status when steroids precede antiviral drugs in patients with COVID-19 pneumonia: A retrospective study
Source: PLoS One. 2021 Sep 2;16(9):e0256977. doi: 10.1371/journal.pone.0256977 (PMC8412353; doi:10.1371/journal.pone.0256977)
Supplement: S4 Table — (DOCX) [file pone.0256977.s007.docx]

**S4 Table.** Comparison between the antiviral-drugs-first group and the steroids-first group in transferred cases.

| Parameter | antiviral-drugs-first group  (n=23) | steroids-first group  (n=16) | P-value |
| --- | --- | --- | --- |
| ICU admission, n (%) | 14 (60.9) | 13 (81.3) | 0.175 |
| Intubation, n (%) | 12 (52.2) | 12 (75.0) | 0.144 |
| ECMO, n (%) | 3 (13.0) | 5 (31.3) | 0.166 |
| Mortality at 30 days, n (%) | 5 (21.7) | 3 (18.8) | 0.820 |

ECMO, Extracorporeal membrane oxygenation. ICU, Intensive care unit.
