# Supplementary material for: Possibility of deterioration of respiratory status when steroids precede antiviral drugs in patients with COVID-19 pneumonia: A retrospective study
Source: PLoS One. 2021 Sep 2;16(9):e0256977. doi: 10.1371/journal.pone.0256977 (PMC8412353; doi:10.1371/journal.pone.0256977)
Supplement: Attachment — Submitted filename: Comments for the authors.docx [file pone.0256977.s009.docx]

**Comments for the authors:**

This was a retrospective study; hence, other treatment modalities could have been different in the two groups, e.g. anticoagulants. Did all or some of the patients receive anticoagulants / Was anticoagulant a part of treatment protocol? The authors need to comment on this. Line no. 171, "..incidence of intubation and ECMO.." should be changed to **".. the duration of intubation and ECMO.."** as per the data presented in table 2.

The following issues may be commented on / added in the discussion section. Along with the duration of symptom onset, clinical evaluation/hypoxia is also an essential factor to decide regarding initiation of steroids in Covid-19 infection. However, today's dilemma is that robust evidence regarding the absolute beneficial role of antivirals in covid-19 is lacking. No specific drugs have been approved for COVID-19 disease to date. (1) Other than the timing of corticosteroid, duration of corticosteroid therapy in covid-19 infection is also an important factor, and as evidenced from the RECOVERY trial, the patients were administered steroid therapy up to 10 days. (2) Also, corticosteroids may potentially have a procoagulant effect in covid-19; hence the addition of anticoagulant therapy may also be considered in the treatment protocol of covid-19. This could have been a potential factor, wherein steroid therapy aggravated the treatment outcomes in the absence of an antiviral, which could potentially have controlled the viremia associated procoagulant effect of the covid-19 infection at an earlier stage of the disease. (3)

The study presents a very logical finding that steroid therapy without the cover of antiviral therapy may be detrimental.

**References:**

1.	Frediansyah A, Tiwari R, Sharun K, Dhama K, Harapan H. Antivirals for COVID-19: A critical review [Internet]. Vol. 9, Clinical Epidemiology and Global Health. Elsevier B.V.; 2021 [cited 2021 Jun 24]. p. 90–8. Available from: https://pubmed.ncbi.nlm.nih.gov/33521390/

2.	Dexamethasone in Hospitalized Patients with Covid-19 — Preliminary Report. N Engl J Med [Internet]. 2020 Jul 17 [cited 2020 Oct 30]; Available from: https://www.nejm.org/doi/full/10.1056/NEJMoa2021436

3.	Mishra GP, Mulani J. Corticosteroids for COVID-19: the search for an optimum duration of therapy. Lancet Respir Med [Internet]. 2021 Jan 1 [cited 2021 Jan 1];9(1):e8. Available from: https://doi.org/10.1016/S2213-2600(20)30530-0
